# Examining the Effects of Gestational Physical Activity and Hofbauer Cell Polarization on Angiogenic Factors

**DOI:** 10.3390/ijerph20136298

**Published:** 2023-07-04

**Authors:** Alexandra D. Goudreau, Layli Tanara, Velislava Tzaneva, Kristi B. Adamo

**Affiliations:** 1 Faculty of Health Sciences, University of Ottawa, Ottawa, ON K1S 5L5, Canada; 2 Faculty of Sciences, University of Ottawa, Ottawa, ON K1S 5L5, Canada

**Keywords:** physical activity, placenta, Hofbauer cells, macrophage polarization, angiogenesis, pregnancy

## Abstract

While gestational physical activity (PA) has demonstrated health benefits for both birthing parent and fetus, the mechanisms still need to be fully understood. Placental macrophages, or Hofbauer cells (HBCs), comprise a heterogenous population containing inflammatory (CD206-) and anti-inflammatory (CD206+) phenotypes. Similar to other tissue-resident macrophages (TRMs), HBCs are potential mediators of angiogenesis due to their secretion of both pro- and anti-angiogenic factors, including FGF2, VEGF, and SPRY2. While PA is associated with an increase in the proportion of VEGF- and FGF2-producing CD206+ macrophages in other tissues, the phenotypes producing FGF2, VEGF, and SPRY2 in the placenta and the associated relationships with gestational PA have not been studied. Using accelerometry, pregnant participants were classified as physically active or inactive in mid- and late-gestation. Term placenta tissue was collected at delivery and used for Western blotting and immunofluorescence to examine the protein expression of FGF2 and SPRY2, and to localize FGF2 in histological samples, respectively. Primary cultures of HBCs were used to examine the phenotypic differences in FGF2, SPRY2, and VEGF production. While no differences in the placental expression of SPRY2, total FGF2, or high-molecular-weight FGF2 were observed based on PA status, active individuals had significantly reduced levels of low-molecular-weight FGF2. Additionally, HBCs of all polarizations produce VEGF, FGF2, and SPRY2, and can form intercellular junctions and multinucleated giant cells. These findings suggest a possible relationship between PA and HBC-driven angiogenesis, providing an avenue for future exploration.

## 1. Introduction

It has been thoroughly documented in the literature that habitual physical activity (PA) throughout gestation can contribute to the development of a healthy pregnancy and reduce the risk of complications, including pre-eclampsia, gestational hypertension, and gestational diabetes mellitus (GDM) [1]. Individuals who are consistently physically active throughout their pregnancy are also less likely to develop urinary incontinence or experience post-partum weight retention [1]. Through modulating GWG, PA also reduces the risk of delivering by caesarian section, prolonged labour, and complications requiring instrumental delivery interventions with forceps or vacuums [1]. Despite the illustrated benefits of PA throughout pregnancy, the mechanisms through which they arise are not fully understood.

The human placenta is unique, as both a transient and multifunctional organ. Serving as the interface between the gestational parent and developing fetus, it carries nutrients and oxygen to the fetus, removes waste products, and has a protective role by forming an immunological barrier. As the only immune cells in the villous chorion, Hofbauer cells (HBCs) are placenta resident macrophages of fetal origin that remain present throughout gestation [2,3,4]. Similar to other macrophage populations, HBCs can adopt different phenotypes based on their microenvironment [2,5]. Generally speaking, the spectrum of polarization can be divided into classically activated, pro-inflammatory M1 subtypes, and alternatively activated, anti-inflammatory M2 subtypes. M1 macrophages are typically involved in pathogen resistance, while M2 macrophages act as mediators of the inflammatory response and contribute to tissue growth and repair. M2 populations can be further diversified, containing M2a, M2b, M2c, and M2d phenotypes. In keeping with other tissue-resident macrophage populations, HBCs possess heterogenous states of polarization [2,5]. Healthy pregnancies are characterized by a lack of an M1 phenotype, with M2a and M2c (CD206+) HBCs comprising the majority. M2b phenotypes are present in healthy pregnancies; however, greater expression of the M2b phenotype has been associated with detrimental conditions, such as gestational diabetes mellitus (GDM) [6].

**Figure 1:**
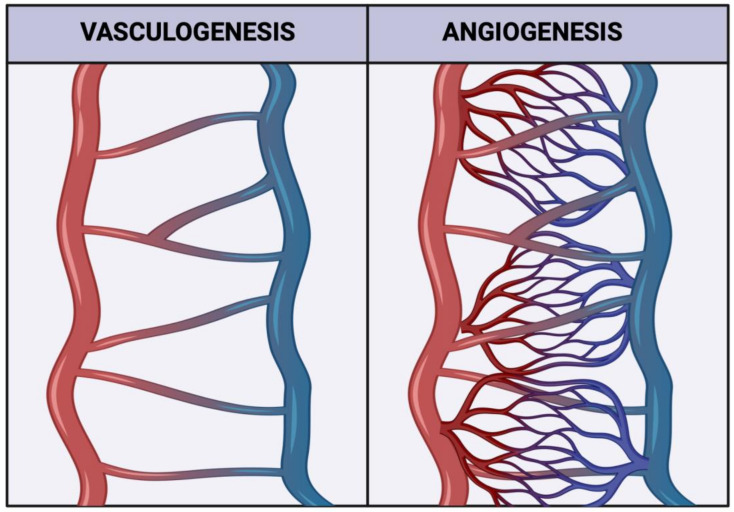
Representative schematic illustrating how both vasculogenesis and angiogenesis contribute to forming a mature vascular network. Created with biorender.com (accessed on 11 September 2022).

HBCs have been shown to play a part in the development of the placental circulatory system. The vasculature of the placenta is special in the fact that it contains two distinct vascular networks. The uteroplacental and fetoplacental circulatory systems carry parental and fetal blood, respectively, to the gestational interface in order to facilitate nutrient and gas exchange [7]. To develop this vasculature, the growing placenta undergoes two critical processes. Vasculogenesis, or the de novo formation of new blood vessels, and angiogenesis, the development of the smaller vessels that form the capillary beds (Figure 1) [8]. HBCs have been implicated in both vasculo- and angiogenesis in the placenta through their secretion of angiogenic factors, such as fibroblast growth factor 2 (FGF2) and vascular endothelial growth factor (VEGF) [5,9]. Interestingly, in recent years, the FGF2-mediated angiogenesis inhibitor Sprouty 2 protein (SPRY2) has also been shown to be secreted by HBCs [10]. Isoforms of FGF2 include high molecular weight (HMW) and low molecular weight forms [11]. HMW-FGF2 is a mitogenic factor in the nucleus, while LMW-FGF2 is secreted to promote angiogenesis [11]. The expression of both pro- and anti-angiogenic factors by HBCs makes them likely regulators of blood vessel development. It is currently unclear if HBC polarization leads to differences in SPRY2 secretion, and while M2 macrophages have been shown to secrete both VEGF and FGF2, with CD206+ macrophages being the main producers of FGF2 [12], the role of HBC subtypes in the secretion of VEGF and FGF2 has not been well studied. In previous research conducted by our lab, physically active individuals were shown to express higher levels of VEGF [13], and have augmented proportions of CD206+ cells, indicative of an M2a or M2c phenotype [14]. As such, this study aimed to explore which HBC subtypes expressed FGF2, VEGF, and SPRY2, as well as to examine the potential associations between PA and the expression of FGF2 and SPRY2.

## 2. Materials and Methods

### 2.1. Participant Recruitment and Ethical Approval

Pregnant individuals from Ottawa, Ontario, were invited to participate in the PhysicaL Activity and diEtary implicatioNs Throughout pregnAncy (PLACENTA) study. Participants were pre-screened by phone to assess study edibility by the a priori inclusion and exclusion criteria (Table 1). Informed written consent was obtained before enrollment into the PLACENTA study. The PLACENTA study was approved by the Research Ethics Board (REB) of the University of Ottawa (file number: H11-15-29) and conformed to all aspects of the Declaration of Helsinki.

### 2.2. Accelerometry Data Capture

Individuals completed two in-person assessments in mid (24 to 28 weeks gestation) and late (34 to 38 weeks gestation) pregnancy. At the end of each visit, a take-home package was provided that contained an omnidirectional Actical^®^ accelerometer (Philips Respironics, Bend, OR, USA), which was to be worn around the waist during waking hours over a period of seven consecutive days to record periods of free-living PA. A minimum of three completed days with ten or more wear hours per day was required to be included in further analyses [15]. Accelerometry data were analyzed to yield the minutes of PA accumulated over the seven days. As per the Canadian Health Measures Survey procedures, data analysis was performed as previously described [16] using SAS version 9.4. PA status was assessed based on the 2019 Canadian guideline for physical activity during pregnancy recommendation to accumulate a total of 150 min of moderate PA (MPA) each week, or an average of 21.4 min per day [1]. Participants in the physically active group averaged 21.4 or more minutes per day of moderate-to-vigorous PA (MVPA). Participants in the physically inactive group averaged less than 21.4 MVPA minutes per day. The most and least physically active participants were chosen for future analysis.

### 2.3. Collection of Term Placenta Samples

Within an hour of vaginal delivery or caesarian section, term placentae were collected and the umbilical cord and chorioamniotic membranes were removed. All sampling was performed on ice and avoided areas of excess calcifications, abruptions, or necrosis. Tissue samples of approximately 2.5 cm^3^ were taken from both central and peripheral cotyledons and then divided into smaller pieces of approximately 1 cm^3^. Dissected samples were placed into cryovials and flash-frozen in liquid nitrogen for transport, then stored at −80 °C. To isolate placenta protein lysate, frozen samples were powdered on ice and homogenized with the Powergen 125 homogenizer (Fisherbrand, Pittsburg, PA, USA) in radioimmunoprecipitation buffer (BioRad, Hercules, CA, USA). Lysate underwent 1000× *g* centrifugation at 4 °C for 10 min to remove debris. In addition to tissue samples, full-thickness histological samples were taken from the fetal side of healthy cotyledons. Sections were fixed in formalin for 48 h before being embedded in paraffin blocks.

### 2.4. Western Blotting

The total protein content of placenta tissue lysate was determined using a DC protein assay (Bio-Rad Laboratories, Mississauga ON, Canada). Thirty and forty μg of protein were loaded onto Mini-PROTEAN^®^ TGX gel (Bio-Rad Laboratories, ON, Canada) for Western blot analysis of SPRY2 and FGF2 protein expression, respectively. The protein content was resolved using SDS-page electrophoresis in reducing conditions at 150 volts for 1 h. Following electrophoresis, the protein was transferred onto a polyvinylidene difluoride (PVDF) membrane (Bio-Rad Laboratories, ON, Canada) and blocked with 5% non-fat dry milk in tris buffered saline solution (TBST) for 1 h at room temperature (RT). The membranes were incubated at 4 °C overnight in primary antibodies diluted at 1:1000 in 5% milk. The primary antibodies used were rabbit monoclonal recombinant anti-FGF2 antibody (1:1000, ab92337, Abcam, Toronto ON, Canada) and rabbit monoclonal recombinant anti-SPRY2 antibody (1:1000, ab180527, Abcam). Thereafter, blots were washed with TBST before a 1 h incubation at RT with the horseradish-peroxidase conjugated secondary antibody, goat anti-mouse IgG (1:5000, Bio-Rad Laboratories, ON, Canada). After washing with TBST, the blots were developed with Clarity ECL Western Substrate (Bio-Rad Laboratories, ON, Canada) and imaged using the ChemiDoc™ XRS+ Imaging System (Bio-Rad Laboratories, ON, Canada). Amido black at a 1% concentration was used to permanently stain the blots for total protein lane quantification. Densitometric analysis (Image J, Bio-Rad Laboratories, ON, Canada) was used to quantify the relative expression of the target band and total protein. Protein expression of FGF2 and SPRY2 were standardized to total participant pooled lysate samples.

### 2.5. Hofbauer Cell Culture

Primary cultured isolated HBCs were purchased from the Amnion Foundation (Winston-Salem NC, USA). Preliminary experiments were conducted to determine the optimal culture time. As per manufacturer instructions, cells were cultured for a maximum of 7 days before examination. It was determined that by reducing the total culture time to 5 days, cell viability at the end of the period was moderately improved.

**Figure 2:**
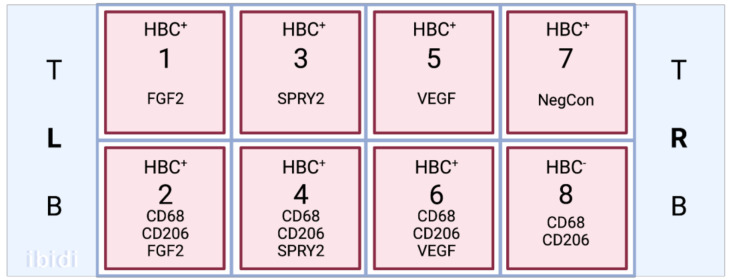
A schematic of the iBidi slide layout in relation to HBC culture and application of primary antibodies.

Cells were seeded in wells 1–7 (Figure 2) on iBidi 8-well slides (WI, USA), while well 8 served as a contamination control. Cultures were maintained in Gibco RPMI Media (Thermo Fisher Scientific, Nepean, ON, Canada), supplemented with 5% fetal bovine serum (FBS) and 25 mM HEPES, at 37 °C and 5% CO. To mimic the chronic physiological normoxic conditions of the term placenta, an oxygen concentration of 8% was used [17]. HBC complete media was filtered using a Stericup quick release-VP sterile vacuum filtration system with a 0.1 μm pore size to mitigate the risk of mycoplasma contamination. For all experiments, cells were seeded and incubated overnight to allow for adhesion. Media was changed at 24 and 72 h post-seed, before fixation and immunostaining at 120 h post-seed.

### 2.6. Immunofluorescence Staining

#### 2.6.1. In Vitro Immunofluorescence Staining

Immunofluorescent staining was performed to localize FGF2, SPRY2, and VEGF within cultured HBCs. After being cultured for 5 days in normoxic conditions, the media were removed from each iBidi well, and cells were washed three times in sterile PBS. The protocol for cell fixation and permeabilization was performed at RT as follows. Cells were fixed with 4% paraformaldehyde diluted in sterile PBS for 15 min, then washed three times with ice-cold PBST. For permeabilization, a 10 min incubation in 0.1% Triton X-100 in PBS was performed before washing again in PBS. Before immunostaining, fixed cells were incubated with 1% BSA in PBST (PBS + 0.1% Tween 20) for 30 min to block unspecific binding of the antibodies. Primary antibodies for CD68 (mouse monoclonal; Abcam ab201973) and CD206 (rabbit polyclonal; Abcam ab64693) were diluted in PBST with 1% BSA at 4 μg/mL and 2 μg/mL, respectively. Wells 2, 4, 6, and 8 (Figure 2) were incubated in the diluted antibodies overnight at 4 °C, while wells 1, 3, 5, and 7 were incubated in PBST. The following day, all wells were washed with PBST and incubated in the dark for 1 h with a secondary antibody solution composed of 1:1000 dilutions of Alexa 488 goat anti-mouse and Alexa 594 goat anti-rabbit antibodies (Thermofisher, Ottawa ON, Canada). To visualize angiogenic factors in relation to cultured HBCs, a 1:200 dilution of rabbit monoclonal recombinant anti-FGF2 antibody (ab92337, Abcam) was added to wells 1 and 2, a 1:100 dilution of rabbit monoclonal recombinant anti-SPRY2 antibody (ab180527, Abcam) was added to wells 3 and 4, and a 1:100 dilution of mouse monoclonal anti-VEGF primary antibody (NB100-664, Novus Biologics, Centennial CO, USA) was added to wells 5 and 6. PBST was added to wells 7 and 8 to prevent cell drying. The cells were incubated overnight in the dark at 4 °C, then washed three times with PBST. A 1:1000 dilution of the secondary antibody Alexa 647 goat anti-rabbit or anti-mouse (Thermofisher) was added to the applicable wells, determined by the species of the corresponding primary antibody, and allowed to incubate for 1 h in the dark at RT. After the slides were rinsed again three times in PBST, Prolong Gold with DAPI was applied to the wells, and the slide was wrapped in paraffin to prevent drying before cell imaging. The application of primary antibodies was omitted in negative controls. Slides were imaged using a confocal microscope (LMS880 AxioObserver Z1, Zeiss, Toronto, ON, Canada) and Airyscan processing.

#### 2.6.2. In Vivo Immunofluorescence Staining

Immunofluorescent staining was again performed to localize FGF2 in relation to HBCs in collected placenta tissue. Formalin-fixed paraffin-embedded (FFPE) tissue was processed into 4 μm thick sections and mounted on slides by the Louise Pelletier Histology Core at the University of Ottawa. Slides were deparaffinized and rehydrated using xylene and ethanol in a graded series of dilutions and rinsed in double-distilled water. Sodium citrate buffer (10 mM pH 6.0) was utilized during heat-induced epitome retrieval before the tissue was permeabilized in 0.2% Tiron-X in TBS for 20 min at RT. To block non-specific binding and to quench tissue autofluorescence, the slides were incubated at RT for 1 h in 10% bovine serum albumin, then for 10 min in 0.1% Sudan Black in 75% EtOH (*w*/*v*). Primary antibodies for CD68 (mouse monoclonal; Abcam ab201973) and CD206 (rabbit polyclonal; Abcam ab64693) were diluted in TBST at 4 μg/mL and 2 μg/mL, respectively. Slides were incubated in the diluted antibodies overnight at 4 °C. Once the incubation was complete, slides were washed in TBST and then incubated in the dark for 1 h with a secondary antibody solution containing a 1:250 dilution of Alexa 488 goat anti-mouse and a 1:1000 dilution of Alexa 594 goat anti-rabbit antibodies (Thermofisher). Slides were rinsed three times for five minutes in TBST then incubated overnight at 4 °C in a 1:1000 dilution of rabbit monoclonal recombinant anti-FGF2 antibody (1:200, ab92337, Abcam). Following the overnight incubation, a 1:1000 dilution of the secondary antibody Alexa 647 goat anti-rabbit (Thermofisher) was applied for 1 h at room temperature. After the slides were rinsed again three times for five minutes in TBST, Prolong Gold with DAPI was used to mount the slides, and the edges were sealed with nail polish. The application of primary antibodies was omitted in negative controls. Slides were imaged using a fluorescent microscope (AxioObserver M2, Zeiss) equipped with blue (excitation 390/22 nm, emission 460/50 nm), green (excitation 470/40 nm, emission 525/50 nm), and red (excitation 560/40 nm, emission 630/75 nm) filters to visualize the cells of interest.

### 2.7. Statistical Analysis

GraphPad Prism software (version 9.0.0, GraphPad Software Inc., La Jolla, CA, USA) was used for statistical analyses of data, which are presented herein as mean ± standard deviation. The normality of data was tested with Shapiro–Wilks tests. Unpaired *t*-tests or Mann–Whitney U tests, where appropriate, were used to analyze participant demographics, Western blot protein expression, and cell counts between active and inactive participants. Pearson correlations were also used to analyze the protein expression and cell counts in comparison to the average minutes of MVPA/day accumulated by each participant. Statistical significance was defined as *p* < 0.05.

## 3. Results

### 3.1. Participant Demographics

Maternal demographic information and newborn outcomes are described in Table 2 according to PA status throughout gestation. By study design, physically active participants had significantly higher MVPA (min/day) than their inactive counterparts in both mid and late gestation (*p* < 0.001). Maternal age, height, pre-pregnancy weight, or pre-pregnancy BMI did not differ significantly between active and inactive groups. On average, neonates born to physically inactive participants had significantly higher birth weights and lengths when compared to their counterparts born to active individuals.

### 3.2. Expression of FGF2 and SPRY2 in Term Placenta

**Figure 3:**
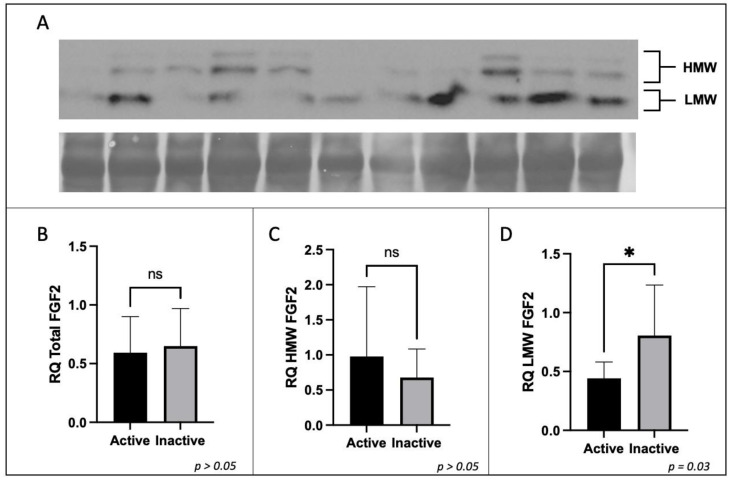
Expression of FGF2 protein by Western immunoblotting in term placenta (2:1 ratio of tissue from the central and peripheral regions of the placenta, respectively) from physically active (*n* = 10) and inactive (*n* = 7) participants. A representative immunoblot for (**A**) FGF2 is shown. The corresponding semi-quantitative densitometric analysis is shown for (**B**) total FGF2 expression, (**C**) high molecular weight FGF2 expression, and (**D**) low molecular weight FGF2 expression. All data are represented as mean ± SD. * *p* ≤ 0.05. ns: not significant.

**Figure 4:**
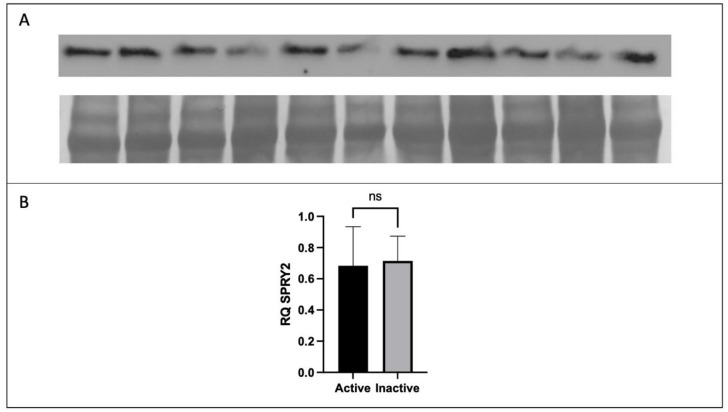
Expression of SPRY2 protein by Western immunoblotting in term placenta (2:1 ratio of tissue from the central and peripheral regions of the placenta, respectively) from physically active (*n* = 10) and inactive (*n* = 7) participants. A representative immunoblot for (**A**) SPRY2 is shown. The corresponding semi-quantitative densitometric analysis is shown for (**B**) SPRY2 expression. All data are represented as mean ± SD. * *p* ≤ 0.05. ns: not significant.

The protein expression of placental CD68 and CD206 were analyzed to determine the difference in expression between active and inactive participants by *t*-test or Mann–Whitney U test where applicable. No significant difference in the protein expression of total FGF2, HMW FGF2, or SPRY2 was found between active and inactive women (*p* > 0.05). However, the protein expression of LMW FGF2 was significantly lower in active women when compared to their inactive counterparts (Figure 3 and Figure 4).

### 3.3. Expression of FGF2 in Term Placenta

**Figure 5:**
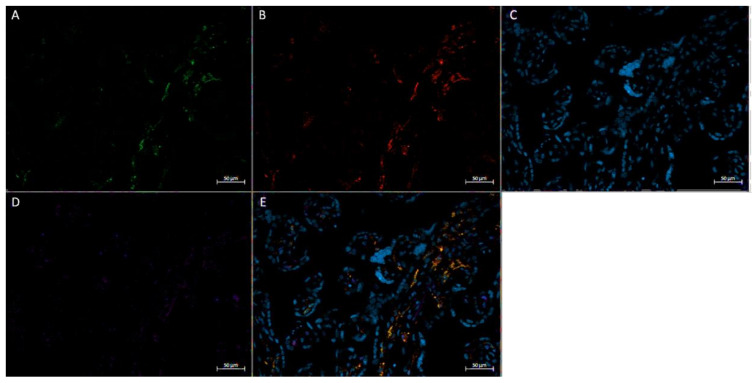
Immunofluorescence staining of CD68, CD206, and FGF2 of a physically active participant. FFPE placenta tissue slide imaged with an Axio Imager M2 epifluorescent microscope. (**A**) CD206; (**B**) FGF2; (**C**) DAPI (cell nuclei); (**D**) CD68; (**E**) merged image. Scale bar is 50 μm.

**Figure 6:**
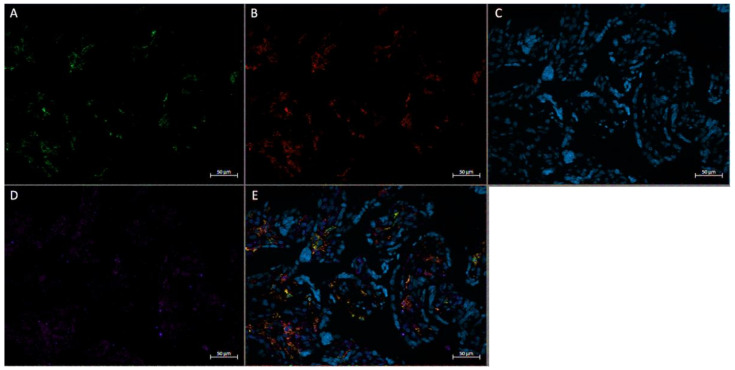
Immunofluorescence staining of CD68, CD206, and FGF2 of a physically inactive participant. FFPE placenta tissue slide imaged with an Axio Imager M2 epifluorescent microscope. (**A**) CD206; (**B**) FGF2; (**C**) DAPI (cell nuclei); (**D**) CD68; (**E**) merged image. Scale bar is 50 μm.

In vivo immunofluorescence studies showed the localization of FGF2, the pan-macrophage marker CD68, and M2a/M2c macrophage marker CD206 in both active (Figure 5) and inactive (Figure 6) participants. In term placenta, it can be observed that FGF2 is colocalized within cells that express CD68, regardless of the presence of CD206, indicative of FGF2 expression in M2a, M2b, and M2c phenotypes.

### 3.4. Expression of Angiogenic Markers in Cultured Hofbauer Cells

**Figure 7:**
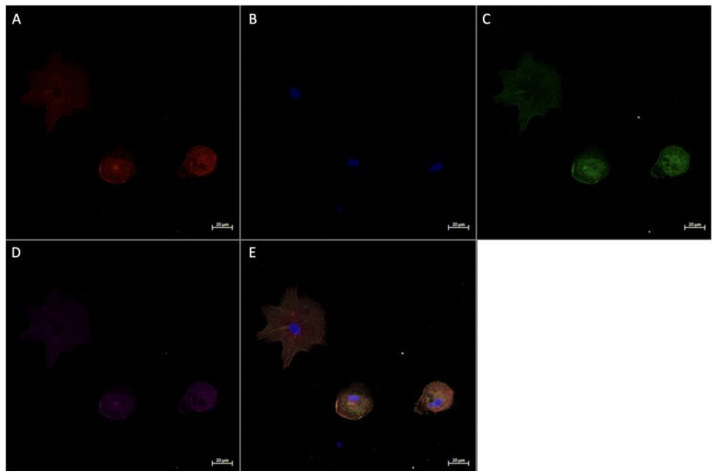
Immunofluorescence staining of CD68, CD206, and FGF2 in cultured Hofbauer cells imaged at 20× magnification with the Zeiss LMS880 AxioObserver Z1 confocal microscope. (**A**) CD206; (**B**) DAPI (cell nuclei); (**C**) CD68; (**D**) FGF2; (**E**) merged image. Scale bar is 20 μm.

**Figure 8:**
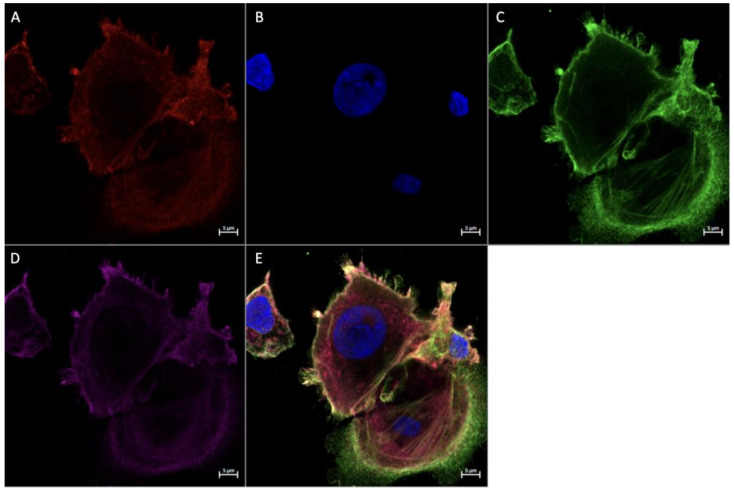
Immunofluorescence staining of CD68, CD206, and FGF2 in cultured Hofbauer cells imaged at 63× magnification with the Zeiss LMS880 AxioObserver Z1 confocal microscope. (**A**) CD206; (**B**) DAPI (cell nuclei); (**C**) CD68; (**D**) FGF2; (**E**) merged image. Scale bar is 5 μm.

**Figure 9:**
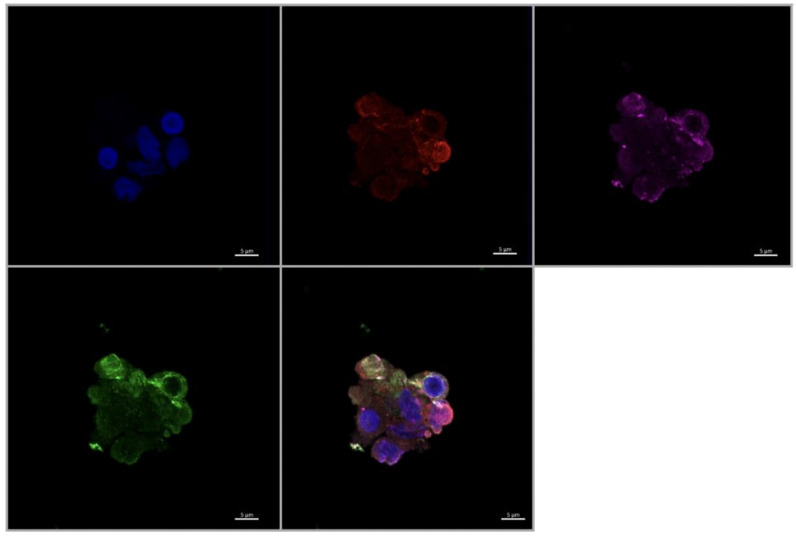
Immunofluorescence staining of CD68, CD206, and VEGF in cultured Hofbauer cells imaged at 63× magnification with the Zeiss LMS880 AxioObserver Z1 confocal microscope. (**A**) CD206; (**B**) DAPI (cell nuclei); (**C**) CD68; (**D**) VEGF; (**E**) merged image. Scale bar is 5 μm.

**Figure 10:**
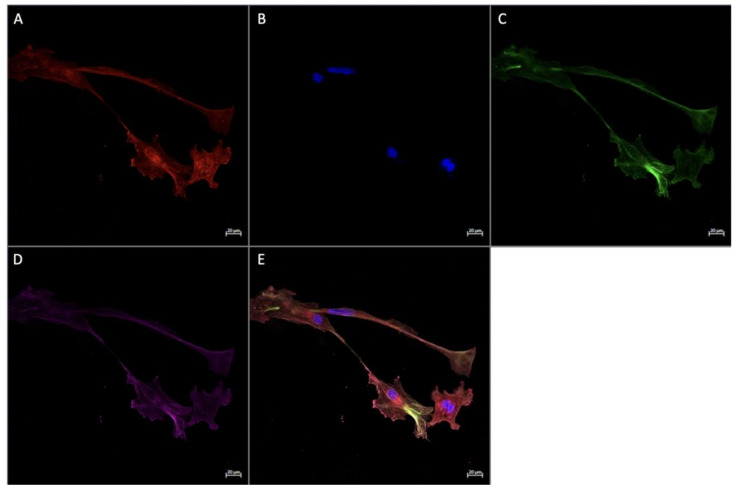
Immunofluorescence staining of CD68, CD206, and SPRY2 in cultured Hofbauer cells imaged at 20× magnification with the Zeiss LMS880 AxioObserver Z1 confocal microscope. (**A**) CD206; (**B**) DAPI (cell nuclei); (**C**) CD68; (**D**) SPRY2; (**E**) merged image. Scale bar is 20 μm.

**Figure 11:**
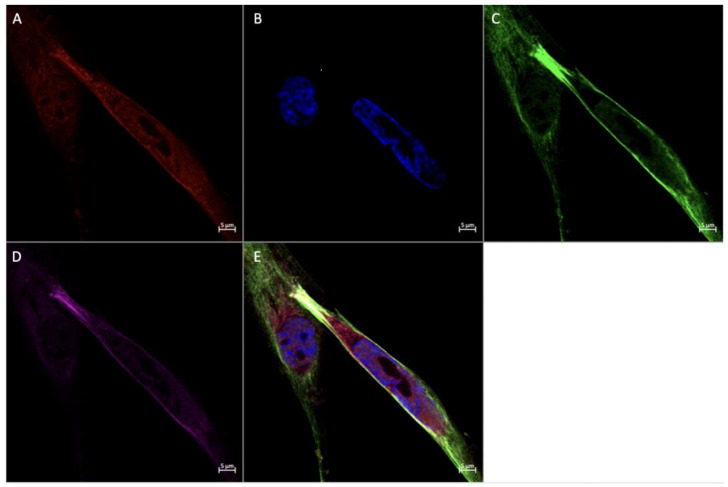
Immunofluorescence staining of CD68, CD206, and SPRY2 in cultured Hofbauer cells imaged at 63× magnification with the Zeiss LMS880 AxioObserver Z1 confocal microscope. (**A**) CD206; (**B**) DAPI (cell nuclei); (**C**) CD68; (**D**) SPRY2; (**E**) merged image. Scale bar is 5 μm.

Consistent with our findings stated herewithin, as well as previous work by our lab [14], it was seen that both FGF2 (Figure 7 and Figure 8) and VEGF (Figure 9) were colocalized within cells expressing CD68 with or without the expression of CD206. In a similar manner to both FGF2 and VEGF, SPRY2 was also found in all CD68+ cultured HBCs (Figure 10 and Figure 11).

Morphologically, HBCs in culture were observed to possess the high degree of adhesive properties that is often observed in macrophage populations. This is demonstrated through macrophage aggregates (Figure 8 and Figure 9) and intercellular connections (Figure 10 and Figure 11).

## 4. Discussion

It has been demonstrated in past research that HBCs express both the pro-angiogenic factor FGF2 and anti-angiogenic factor SPRY2 [3,7,8], marking them as potential contributors to the development of vasculature in the placenta. Recent findings from our lab showed that physically active participants have elevated proportions of CD206+ subtypes (Goudreau, unpublished). In other tissue-resident macrophages, these phenotypes have been identified as the drivers of FGF2 [18], while SPRY2 expression in different macrophage polarizations has not been determined. As such, this novel study aimed to elucidate the role of PA and macrophage polarization in the production of FGF2 and SPRY2 within the placenta. Though there were no observed differences in the protein expression of SPRY2, total FGF2, or HMW FGF2 between active and inactive participants, active individuals had significantly lower levels of LMW FGF2 in term placenta. We also report for the first time that HBCs of all polarizations produce VEGF, FGF2, and SPRY2. Additionally, we observed that HBCs demonstrate the ability to form filopodia and cell aggregates, marking them as potential candidates to develop intercellular junctions and multinucleated giant cells (MGC).

The establishment of a vascular network within the placenta is a crucial process that allows it to act as a multi-organ surrogate for the developing fetus. As the placenta develops, the chorionic villi containing fetal capillaries expand into the intervillous space to intermingle with parental vessels [19]. The transport of gases, nutrients, and waste at this interface allows the placenta to act as the lungs, liver, and kidneys of the fetus, while also providing nutrients from parental circulation [20]. HBCs are a mononuclear phagocytic cell family member, which also includes other tissue-resident macrophages such as osteoclasts, alveolar macrophages, Langerhans cells, and microglia [21,22,23,24]. Such macrophages, including HBCs, have been implicated in the development of vasculature through the expression of pro- and anti-angiogenic factors, including SPRYs, FGFs, and VEGF. While there were significant differences observed in LMW-FGF2 protein expression in the term placenta between active and inactive participants, no differences were seen in the expression of HMW-FGF2, T-FGF2, or SPRY2. A previous study from our lab found that VEGF protein and mRNA levels were significantly higher in active individuals [13]. Interestingly, the opposite effect was associated with LMW-FGF2, with active participants expressing significantly less of the protein. One explanation for this may be that because VEGF-driven angiogenesis results in higher vascular permeability than FGF2, the combination of higher VEGF and lower LMW-FGF2 levels may lead to capillary beds with higher vascular permeability, increasing the efficacy of gas and nutrient exchange [25]. Additionally, in contrast to FGF2, VEGF has anti-inflammatory effects, potentially protecting developing tissues from the implantation-induced inflammation and aiding the subsequent shift to an anti-inflammatory environment for the remainder of gestation [26,27,28].

As for other contributors to the development of placental vasculature, SPRYs are membrane-associated proteins that regulate receptor tyrosine kinase (RTK) signalling [8]. The SPRY homologs act as negative regulators for several RTK-induced pathways, including the mitogen-activated protein kinase (MAPK), P13K/Akt, PLC γ pathways, which are stimulated by growth factors such as VEGF and FGF2 [29,30,31,32,33]. While there were differences between PA groups in FGF2 and VEGF protein levels, SPRY2 remained unchanged based on PA. The literature clearly demonstrates that dysregulations in RTK pathways can have deleterious effects. While underactive pathways lead to insufficient angiogenesis and decreased cell survival [34], overexpression has been linked to proliferative disorders and vascular anomalies [35,36]. Thus, it is possible that SPRY2 expression is highly conserved in order to prevent deleterious dysregulations of RTK-induced pathways, thereby adding a level of protection to the development of the vascular network.

As both CD206+ and CD206- HBCs expressed SPRY2 and FGF2 in in vitro and in vivo models, it is logical to posit that all subtypes contribute to the production of the angiogenic factors, reinforcing the recent focus on HBCs as mediators of placental angiogenesis. The quantity of FGF2 isomers secreted from distinct HBC subtypes in both healthy and complicated pregnancies, as well as the potential significance FGF2 expression has on fetal outcomes, should be explored in future research.

Using immunofluorescence to visualize HBCs in culture, we observed their tendency to interact with each other, demonstrated by the formation of filopodia and cellular aggregates. Interestingly, both aforementioned processes are critical steps in the formation of multinucleated giant cells (MGCs) [37]. It has been shown that M2 macrophages, which form the majority of the HBC population, express chemokine ligand 2 (CCL2) [38]. Under the influence of CCL2, macrophage motility increases and membrane-protruding filopodia are formed [37]. Interacting filopodia form intercellular connections that facilitate the genesis of cell aggregates, within which macrophages adhere and rearrange their cytoskeletons, extracellular membranes, and organelles [37]. In this manner, MGCs are formed. To the best of our knowledge, the study by Kesson et al. (1993) provides the only other evidence of this characteristic in cultured HBCs, defined as placental macrophages of fetal origin and distinct from macrophages in the decidua [39]. Historically, the role of MGCs has been linked to cytopathic effects, such as those seen by viral infections [40]. While the inference that MGC formation is indicative of adverse conditions was consistent with the literature at the time, a recent review by Miron et al. (2018) suggested that MGC formation in and of itself may represent neither a healthy nor detrimental environment, and points to the necessity of characterizing the polarization states of such cells [40]. Further research is needed to characterize the profiles of multinucleated giant HBCs and their associated attributes. This research should include immunofluorescence experiments to identify known fusogens (molecules that fuse membranes) and junction proteins present within the process of MGC formation. Electron microscopy could be used to provide more insight into the morphology of these potential placental structures.

There are inherent strengths and limitations associated with the study. It is strengthened by the objective measurement of PA through accelerometry at multiple gestational time points and following a standardized protocol for placenta collection and sample preparation, including using homogenized tissue from multiple placental locations to account for tissue heterogeneity. We also controlled for maternal age, BMI status, and gestational age at delivery. Although the neonates of physically inactive individuals had statistically higher weights and lengths at delivery, all of the measurements were within the appropriate gestational age (AGA) range. Western blotting and immunofluorescence are limited due to their semi-quantitative or qualitative natures. Though flow cytometric analyses were not possible in this pilot study due to a lack of fresh tissue, these results provide a strong basis for future research to examine polarization differences using fluorescence-activated cell sorting in both in vivo and in vitro models. While ensuring that placenta samples from all participants were obtained at term meant it was possible to control for a potential confounding variable, the interactions between activity, HBCs, and angiogenic factors should be examined at multiple gestational time points. As angiogenesis is a cross-gestational process that occurs as placenta vasculature develops and remodels, the results obtained from term tissue may not be generalizable to other gestational time points. To reduce the risk of secondary antibody bleed-through in immunofluorescence experiments resulting in false positive staining, fluorophores with distinct spectra were selected, and single stain controls of each antibody were performed. As the sample size contained within the study is small and relatively homogenous, it is possible that results may not be representative of the larger population. The observations recorded here should be examined in heterogenous cohorts and larger sample sizes.

## 5. Conclusions

In conclusion, while there were no significant differences in the protein expression of SPRY2, total FGF2, or HMW FGF2 based on participant activity status, LMW was lower in active individuals. Combined with previous data observing increased levels of VEGF in physically active participants, there may be a difference in the mechanism of angiogenesis in the placenta to facilitate optimized nutrient and gas exchange between parent and fetus. Furthermore, HBCs of all polarizations both in vivo and in vitro produce VEGF, FGF2, and SPRY2, which strengthens the previous literature implying the role of placental macrophages in gestational angiogenesis. Finally, the capabilities of HBCs in vitro to form intracellular junctions and MGCs provide points of interest for future research into the possible polarizations of MGCs and the interactions of macrophagic networks. The results contained herewithin should drive further exploration of the roles of HBCs in angiogenesis, and the potential effects of gestational PA.

## Figures and Tables

**Table 1:** Inclusion and exclusion criteria for participation in the PLACENTA study.

Inclusion Criteria	Inactive
Between 18 and 40 years of age Proficiency in English and/or French Gestational age below 28 weeks Stable pre-pregnancy weight (± 5 lbs) Pregnant with a singleton fetus Normal or overweight BMI status (18.5–29.9 kg/m^3^)	Contraindications to physical activity Pre-pregnancy diabetes Untreated thyroid disease

**Table 2:** Study participant maternal demographics and newborn outcomes (*n* = 17). Statistically significant *p*-values (*p* < 0.05) are indicated in bold.

	Active (*n* = 10)	Inactive (*n* = 7)	*p*-Value
**Maternal demographics**			
Maternal age (years)	32.50 ± 3.14	32.29 ± 2.69	0.8854
Height (cm)	165.80 ± 6.77	167.19 ± 8.68	0.7163
Pre-pregnancy weight (kg)	64.44 ± 10.87	67.64 ± 15.87	0.6271
Pre-pregnancy BMI (kg/m^2^)	23.39 ± 2.80	23.94 ± 3.48	0.7219
Gestational age at birth (weeks)	40.47 ± 0.90	41.02 ± 0.49	0.1658
Mid gestation MVPA (min/day)	46.79 ± 10.23	6.44 ± 4.01	**<0.0001**
Late gestation MVPA (min/day)	34.65 ± 9.50	3.34 ± 3.43	**<0.0001**
Newborn outcomes			
Birth weight (kg)	3.31 ± 0.38	3.75 ± 0.23	**0.0169**
Birth length (cm)	50.13 ± 2.46	52.69 ± 1.39	**0.0260**
Sex, *n*			
Male	6	3	
Female	4	4	

## Data Availability

Raw data were generated at the University of Ottawa. Derived data supporting the findings of this study are available from the corresponding author KBA upon request.
